# Establishment and transcriptomic characterization of canine organoids from multiple tissues

**DOI:** 10.3389/fcell.2025.1680376

**Published:** 2025-11-24

**Authors:** Christopher Zdyrski, Vojtech Gabriel, Oscar Ospina, Hannah F. Nicholson, Michael Catucci, Bryan J. Melvin, Hannah Wickham, Dipak Kumar Sahoo, Kimberly Dao, Leeann S. Aguilar Meza, Abigail Ralston, Leila Bedos, William Bastian, Sydney Honold, Pablo Piñeyro, Aleksandra Pawlak, Megan P. Corbett, Eugene F. Douglass, Karin Allenspach, Jonathan P. Mochel

**Affiliations:** 1 SMART Pharmacology, Department of Biomedical Sciences, Iowa State University, Ames, IA, United States; 2 3D Health Solutions Inc., Ames, IA, United States; 3 SMART Pharmacology, Precision One Health Initiative, University of Georgia, Athens, GA, United States; 4 Department of Biostatistics and Bioinformatics, Moffitt Cancer Center, Tampa, FL, United States; 5 Department of Veterinary Clinical Sciences, Iowa State University, Ames, IA, United States; 6 Pharmaceutical & Biomedical Sciences, Institute of Bioinformatics, University of Georgia, Athens, GA, United States; 7 Veterinary Diagnostic Laboratory, Iowa State University, Ames, IA, United States; 8 Department of Pharmacology and Toxicology, Faculty of Veterinary Medicine, Wroclaw University of Environmental and Life Sciences, Wrocław, Poland; 9 Department of Physiology and Pharmacology, University of Georgia, Athens, GA, United States; 10 Department of Pathology, College of Veterinary Medicine, University of Georgia, Athens, GA, United States

**Keywords:** canine (dog), organoids, reverse translational medicine, stem cell, endometrium, lung, pancreas, urinary bladder

## Abstract

**Introduction:**

Organoids are 3-dimensional (3D) stem cell-derived cultures that offer a variety of technical advantages compared to traditional 2-dimensional (2D) cell cultures. Although murine models have proved useful in biomedical research, rodent models often fail to adequately mimic human physiology and disease progression, resulting in poor preclinical prediction of therapeutic drug efficacy and toxicity. An interesting alternative is to use the canine model in research, due to its numerous similarities to humans (shared environment, intact immune system, and development of civilization diseases). The use of canine organoids in drug testing and disease modeling has been limited by the number of models as well as the depth of characterization. Therefore, we believe these types of models can expedite drug testing and create a platform for personalized medicine.

**Methods:**

Here, we report the establishment, maintenance, and molecular characterization of six adult-stem cell-derived canine organoid cell lines including endometrium, pancreas, urinary bladder, kidney, lung, and liver from two genetically related canines (B816 and B818). Characterization of these lines was done using multiple techniques including immunohistochemistry (UPKIII, TTF-1) and bulk RNA-seq. Furthermore, scRNA-seq was utilized on a subset of the organoids to identify organoid specific transcriptomic signatures including lung, pancreas, kidney, and bladder.

**Results:**

In total, six tissues and organoid lines from each donor were characterized, allowing for a unique, multi-organ comparison between these two individuals and identification of specific cell types within the organoids. Bulk RNA-seq revealed tissue-specific transcriptomic profiles, with organoids enriched in proliferation-related genes and tissues enriched in inflammation-related genes. Principal component analysis showed organ-based clustering, while scRNA-seq identified diverse epithelial subtypes.

**Conclusion:**

These organoids begin to establish a platform for reverse translational research, reducing reliance on live animal testing. By leveraging genetically related donors, it highlights tissue-specific variations, facilitating applications in personalized medicine, disease modeling, and pharmacology to bridge veterinary and human research gaps.

## Introduction

Numerous *in vitro* models are used as preclinical biological and pharmacological research tools (Hickman et al., 2014). Of the *in vivo* models, mice are extensively used in biomedical research due to their cost-effectiveness, fast-growing nature, and availability of genetic mutants (Kim et al., 2020). However, differences in diet, living environment, circadian rhythm, and the short lifespan are among the issues limiting the translational relevance of rodent models (Perlman, 2016). Although the murine model has proven effective in a variety of biological research areas, rodents frequently fail to adequately mimic human physiology and disease progression, hence compromising their predictive performance in preclinical pharmaceutical research; therefore, the usage of additional and complementary models is warranted (Gordon et al., 2009; Wang J. et al., 2018). Due to some of these challenges, approximately ninety percent of experimental drugs fail to make the transition from discovery to successful clinical trials, emphasizing the need for alternative screening methods (Mochel et al., 2018; Van Norman, 2016; Shalev, 2006).

The reverse translational paradigm, in which data from human clinical research might aid in the development of veterinary therapeutics and *vice versa*, is garnering a growing amount of interest (Schneider et al., 2018). Dogs share similar lifestyles and diets with their owners due to the close relationship between dogs and humans, often including a sedentary lifestyle and an increased risk of developing obesity (Chandler et al., 2017). The longer lifespan of dogs over that of mice predisposes dogs to develop analogous chronic diseases to humans, including diabetes mellitus (Adin and Gilor, 2017), ocular diseases (Sebbag et al., 2018; Sebbag et al., 2019), inflammatory bowel disease (Chandra et al., 2019; Jergens and Simpson, 2012; Kopper et al., 2021), congestive heart failure (Mochel and Danhof, 2015; Mochel et al., 2019; Silva and Emter, 2020), cancers (Knapp et al., 2020), and cognitive dysfunction (Ozawa et al., 2019), among others (Wang J. et al., 2018). Therefore, in addition to being used as a large animal model for preclinical drug safety assessment, dogs are also emerging as a translatable model for demonstrating proof-of-concept efficacy studies, particularly in the field of oncology (LeBlanc et al., 2016; Schaefer et al., 2016). While dogs excel as a model in many applications compared to rodents, they come with their own challenges in the form of expensive housing and ethical concerns about using live dogs in research. Dogs are recognized as companion animals in western countries, and there are ongoing worldwide initiatives to limit their use in research through the 3Rs (*Reduce*, *Replace*, *Refine*) principles (Hasiwa, 2011; Park et al., 2024; Russell and Burch, 1959).

Conventional pharmacology research involves using 2D cell culture and animal testing prior to human clinical trials (Brancato et al., 2020). Ultimately, additional research is warranted to identify alternative *in vitro* models that can more accurately replicate human physiology and reduce animal use. A potential solution that provides more access to the canine model while decreasing reliance on live animal use lies in organoid technology; however, currently, there is a lack of canine organoid models compared to other major biomedical species to accurately depict and study various drugs, diseases, and biological phenomena. The use of 3D organoids in the screening stage of drug discovery could drastically reduce the use of live animals for drug development (Mollaki, 2021).

Organoids are 3D self-organized, miniature, and simplified versions of organs *in vitro*. These organoids can be created from embryonic, induced pluripotent, or adult stem cells. Adult stem cells can self-renew, differentiate into multiple cell types, and are genomically stable over multiple passages (Fatehullah et al., 2016; Huch and Koo, 2015; Huch et al., 2015). Unlike traditional 2D cell lines, organoids grow in a 3D extracellular matrix, allowing for the recreation of more realistic tissue architecture and thus physiological responses (Gopallawa et al., 2024; Hynds and Giangreco, 2013). Furthermore, organoids can be used in both basic and applied biomedical research, including the study of personalized medicine (Bartfeld and Clevers, 2017; Kaushik et al., 2018), regenerative medicine (Sampaziotis et al., 2021), genetic manipulation (Artegiani et al., 2020; Takeda et al., 2019), genetic disorders, cancers, and infectious diseases (Kim et al., 2020; Nantasanti et al., 2015; Usui et al., 2017; Zhou et al., 2018). While human organoids are a valuable research tool in the biomedical field, they come with limitations. Public concern plays a key role in tissue sampling from human patients (Lehmann et al., 2019). Ethical concerns for the use of human-derived organoids include chimeric research and genetic editing of organoids derived from patients (Munsie et al., 2017). Although a growing number of studies have characterized cell populations using transcriptomic data across human organs, there is a lack of similar studies in other models (Jones et al., 2022).

Our laboratory previously demonstrated successful culture of the first canine intestinal organoids from healthy and diseased tissues and demonstrated their translational potential for human medicine (Chandra et al., 2019). Additionally, canine urinary bladder cancer organoids were previously described and exposed to anticancer drugs to describe their potential role in research and precision medicine (Elbadawy et al., 2019). Normal canine lung organoids have briefly been described and used as a comparison to lung adenocarcinoma organoids (Shiota Sato et al., 2023). Canine liver-derived organoids have been previously described from both normal and *COMMD1*-deficient dogs and were cultured to model copper storage disease, which is also known as Wilson’s disease in humans (Nantasanti et al., 2015).

This report describes for the first time the successful establishment of canine endometrium organoid lines (potentially relevant for implantation studies and investigation of endometrial cancers), along with pancreas, kidney, lung, urinary bladder, and liver from the same dogs. By comparing six tissue-specific organoid lines obtained from two genetically related donors (B816 and B818), this study aims to acquire insight into tissue specific transcriptomic expression differences in organoids and their corresponding tissues. Preliminary analyses using bulk RNA sequencing (bulk RNA-seq), immunohistochemistry, and single-cell RNA sequencing (scRNA-seq) on a subset of samples, were used to characterize the relationships between organoid cell lines. In addition to this characterization, we compared related individuals across organs in this preliminary analysis, thus preparing these organoids to be utilized and tested in a variety of biomedical applications and functional assays further reducing the usage of live animal testing.

## Results

### Organoid expansion

**FIGURE 1:**
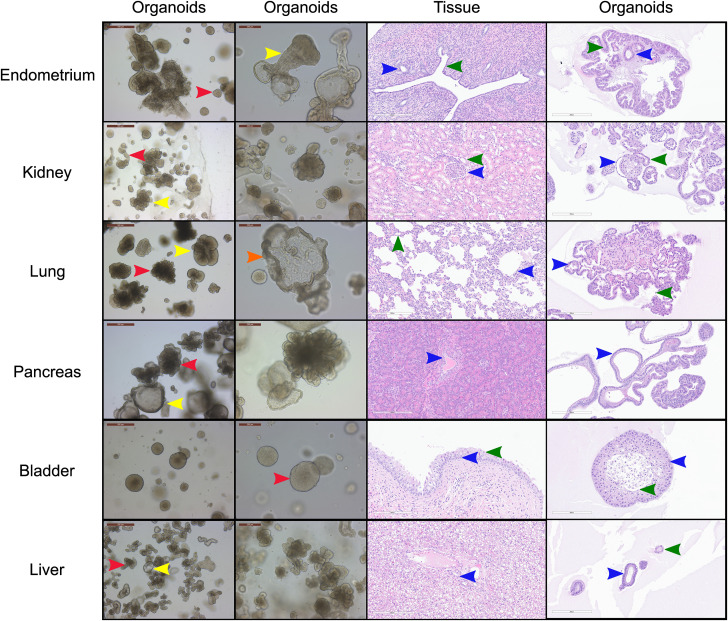
Morphological and histological characterization of canine organoid lines derived from a single donor. Brightfield and hematoxylin & eosin (H&E) images and approximate proportions of the cultures for the six organoid cell lines derived from the uterus (red = 80%, yellow = 20%), kidney (red = 40%, yellow = 60%), lung (red = 60%, yellow = 35%, orange = 5%), pancreas (red = 50%, yellow = 50%) and urinary bladder (red = 100%) of canine individual B816 prior to thawing. Red, yellow, and orange arrows indicate distinct morphologies in each organoid line while blue, green, and black arrows indicate similar histological areas of the organoids and tissues. Structures identified in tissues with histological similarities in organoids were found in endometrium (blue = glandular epithelial cells, green = endometrial epithelial cells), kidney (blue = parietal cells, green = distal tubule), lung (blue = cuboidal epithelial cells, green = alveolar type-1 cells), pancreas (blue = interlobular ducts), urinary bladder (blue = basal cells, green = umbrella cells), and liver (blue = cholangiocyte, green = other observed morphology) organoids. Brightfield images of organoid cultures were captured using a Leica Dmi1 microscope. H&E images were captured from whole image scanned slides using ImageScope software. Scale bars are provided in μm.

Organoid cell lines were successfully established from six organs, including the uterus, lung, pancreas, kidney, urinary bladder, and liver, from two female canine individuals. Notably, the liver and pancreas cultures expanded quickly (Supplementary Figure S1), while endometrium organoids often contained fibroblasts within the culture. Organoids were cultivated simultaneously, with growth progression and passage number reported in Supplementary File S1. Several of the canine organoids displayed a variety of distinct morphological phenotypes characterized via light microscopy and H&E staining (Figure 1). Epithelial origin of the canine organoids was confirmed with positive pan-cytokeratin (PanCK) immunohistochemistry (IHC) and negative smooth muscle actin (SMA) IHC staining, while tissues were positive for both PanCK and SMA (Supplementary Figure S2), alongside negative controls (Supplementary Figure S3). Finally, four of the organoid lines (bladder, kidney, lung, and pancreas) derived from B816 had their thawing potential tested after 3 years in liquid nitrogen storage and were able to be re-expanded prior to scRNA-seq (Supplementary Figure S4).

### Morphological and histological characterization of canine organoid lines

#### Uterus

**FIGURE 2:**
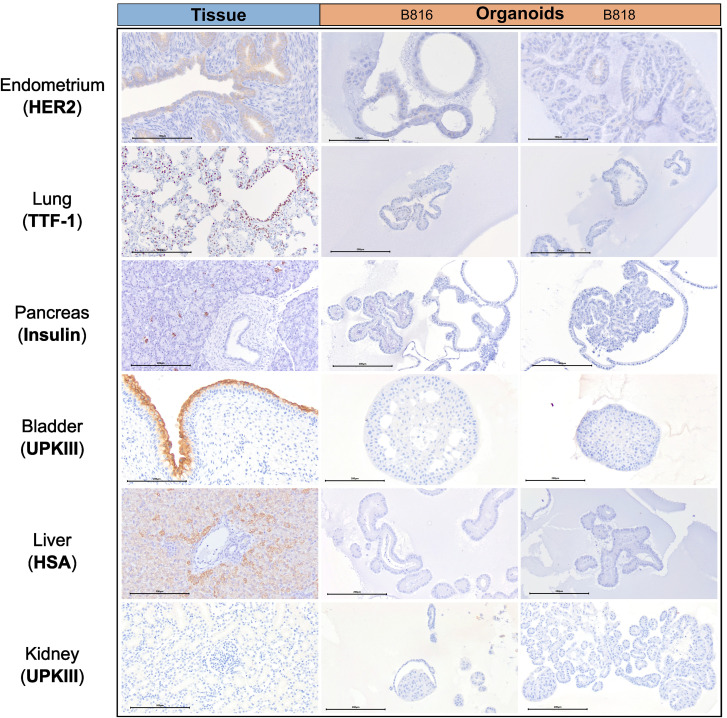
Immunohistochemistry comparison of tissues and organoids. Immunohistochemistry staining of a representative tissue and organoids from both donors, B816 and B818. Antibodies for HER2, TTF-1, Insulin, UPKIII, and HSA were used. Scale bars for each image are displayed in μm.

**FIGURE 3:**
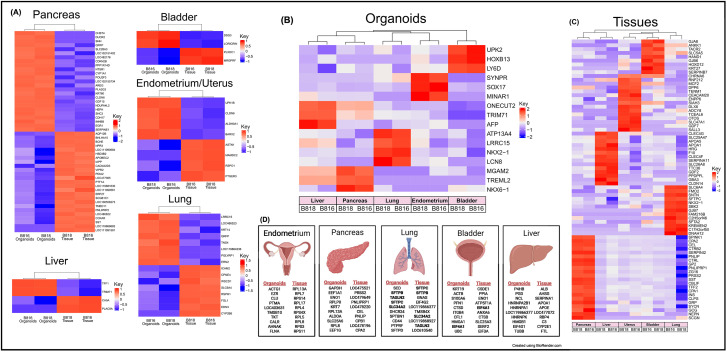
Expression of RNA and identification of tissue-specific markers for both organoids and tissues. **(A)** RNA heatmaps of the differentially expressed (DE) genes (FWER <0.05) between tissues and organoids of the same organs. Tissue-specific markers were identified across the five tissues for both **(B)** organoids (FWER <0.05) and **(C)** tissues (FWER <0.05). Upregulated expression is red, white is neutral, and blue represents suppressed expression. **(D)** The 10 most highly expressed tissue-specific genes from two genetically related donors for both organoids and tissues, as well as genes in common between organoids and tissues are denoted in bold.

**FIGURE 4:**
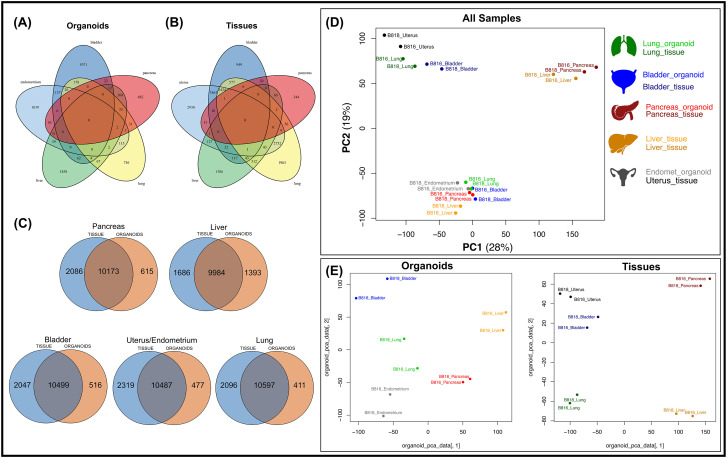
Comparison of mRNA expression similarity between organoids and tissue samples for each organ. Venn diagrams of genes expressed from both donors (B816 and B818) comparing **(A)** organoids and **(B)** tissues from the same organs. **(C)** Venn diagrams showing the comparison of mRNA expression between organoids and tissues from each organ. **(D)** Principal component analysis (PCA) plots of mRNA expression across all organoid and tissue samples. **(E)** PCA plots for either organoid or tissue samples. Tissue types are color coded in the legend.

A subset of endometrial organoids formed a tubular structure on brightfield microscopy during culture, while others remained cystic (Figure 1). There were two morphologically distinct populations including those that resemble endometrial epithelial cells and glandular epithelial cells (Figure 1). IHC for human epidermal growth factor receptor 2 (HER2) seemed lightly positive and in some areas localized to the cell membrane, which we interpret as specific staining in the endometrium organoids (Figure 2). The glandular morphology was supported by the bulk RNA-seq data which indicated that SRY-box transcription factor 17 (*SOX17*) was upregulated in endometrium organoids compared to tissue (Figure 3B). Uroplakin Ib (*UPK1B*) was upregulated in endometrium organoids compared to uterus tissues (Figure 3A). The top endometrium-specific genes for organoids included Actin gamma 1 (*ACTG1*), Clusterin (*CLU*), and Prothymosin alpha (*PTMA*), whereas uterine tissues showed high expression of multiple ribosomal proteins (Figure 3D). Regarding endometrium-specific genes, intra-organoid comparison revealed 1,039 unique genes (Figure 4A), uterine intra-tissue comparisons revealed 2,930 unique genes (Figure 4B), and 10,487 genes were expressed in both organoids and tissues (Figure 4C).

#### Pancreas

**FIGURE 5:**
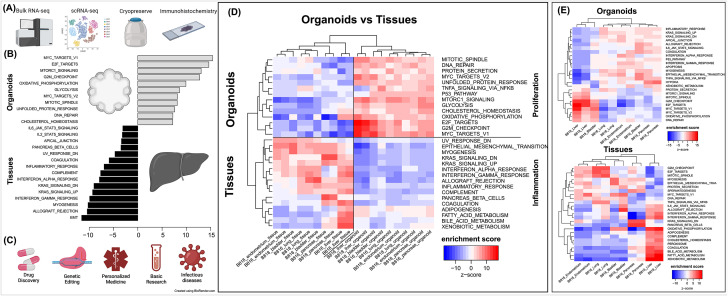
Characterization of organoids, pathway enrichment analysis, and potential biomedical applications. **(A)** Initial characterization and preservation methods used for the intra-donor derived canine organoid cell lines. **(B)** A list of the top pathways when comparing organoids to tissues. **(C)** A list of potential biomedical applications for canine organoid models. **(D)** Analysis of proliferation and inflammation pathways shown across all organoids and tissues. **(E)** Enriched pathways for either organoids or tissues (red = upregulated, blue = downregulated).

**FIGURE 6:**
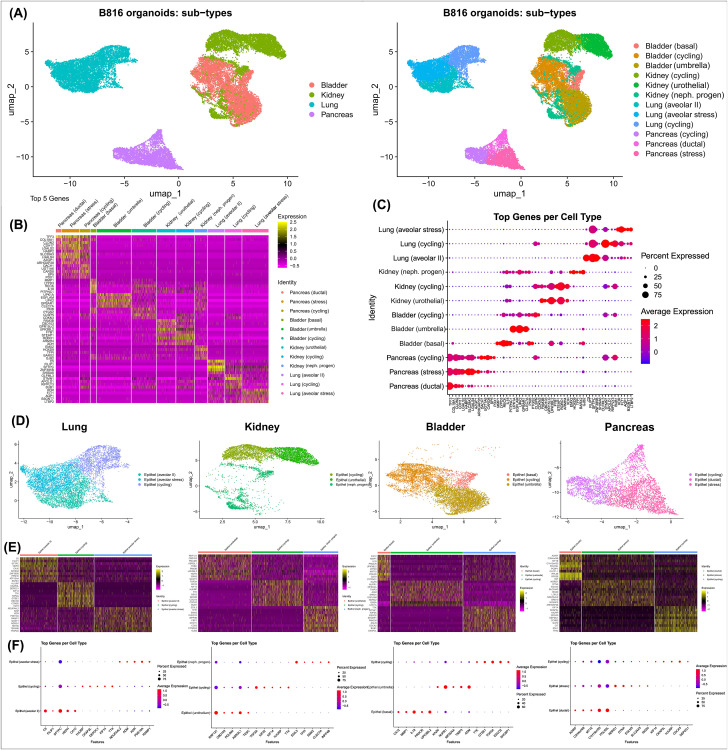
Identification of cell types present in organoids using scRNA-seq. Identification of cell clusters using scRNA-seq for canine organoids derived from the pancreas, kidney, lung, and urinary bladder after thawing. **(A)** Annotated UMAPs displaying the four organoid types compared, as well as the distinct cell clusters (colored) identified in canine organoids. **(B)** Heatmap identifying the top 5 markers for each cluster (upregulated = yellow and downregulated = pink, with respect to each other), expressed as average log2Fold Change. **(C)** Expression of the top 5 genes per cluster. The dot size depicts the percentage of cells in a class and dot color corresponds to the average expression level across all cells within a class (red = higher transcription, blue = lower transcription). **(D)** Annotated UMAPs displaying the four individual organoid types and their relevant cell clusters (colored). **(E)** Heatmaps identifying the genes expressed in each cluster. **(F)** Expression of the top 5 genes per cluster. The dot size depicts the percentage of cells in a class and dot color corresponds to the average expression level across all cells within a class (red = higher transcription, blue = lower transcription).

Light microscopy consisted of two phenotypes, one with a cystic morphology and another of a flowering organoid (Figure 1). Histologically, the culture mainly consisted of cuboidal to low columnar cells resembling intralobular and interlobular ducts (Figure 1). Insulin IHC of pancreatic-derived organoids was negative (Figure 2). Genes upregulated in organoids compared to tissue with bulk RNA-seq included Dual oxidase 2 (*DUOX2*), Pyroglutamylated Rfamide peptide (*QRFP*), Cadherin 17 (*CDH17*), and Early growth response 1 (*EGR1*) (Figure 3A). Notably, Somatostatin (*SST*) expression was reduced in organoids suggesting our culture does not contain a significant number of neuroendocrine delta cells (Figure 3A). However, maltase-glucoamylase 2 (*MGAM2*) and NK6 homeobox 1 (*NKX6-1*) were upregulated in organoids (Figure 3B). Upregulated genes in pancreas tissues included insulin (*INS*), glucagon (*GCG*), and multiple markers characteristic of pancreatic acinar cells (Figure 3C). One of the most highly expressed pancreas-specific genes in the organoids included cytokeratin 7 (*KRT7*). Regarding pancreas-specific genes, intra-organoid comparison revealed 482 unique genes (Figure 4A), intra-tissue comparison revealed 344 unique genes (Figure 4B), and 10,173 genes were expressed in both organoids and tissues (Figure 4C). In addition to the analysis discussed (Figure 5A), scRNA-seq of the pancreas organoids was carried out and there were ∼5,073 cells, 49,832 mean reads per cell, and 4,210 median genes per cell. A total of 3 distinct cell clusters by UMAP were identified as ductal cells, cycling epithelial cells, and stressed epithelial cells (Figure 6A). Ductal cells had high expression of *TFF3* and *CLDN2* (Figure 6B).

#### Kidney

The kidney organoids prior to freezing had two distinct phenotypes, one resembling an organoid mass and the other resembling tubular structures (Figure 1). H&E staining identified multiple organoids containing structures resembling the morphology of glomeruli, Bowman’s capsules, and tubules (Figure 1). Interestingly, the kidney organoids had a simpler morphology under brightfield after thawing of the organoids, which is consistent with their appearance in H&E. Slight UPKIII staining identified some urothelial cells in the kidney organoids only after thawing of the organoids (Supplementary Figure S4). In the kidney organoids, there were ∼6,001 cells, 69,536 mean reads per cell, and 4,426 median genes per cell. A total of 3 distinct cell clusters by UMAP were identified as urothelial cells, nephron progenitor-like cells, and cycling epithelial cells (Figure 6A). The urothelial cluster contained cells having a urothelial-like secretory epithelium signature including those with high expression of *UPK3BL2* and *GCNT1*, while nephron progenitor-like cells expressed *GJB2* and *EMX2* (Figure 6B).

#### Lung

The lung organoids had three distinct phenotypes, with flowering differentiated organoids and bulbous organoids constituting most of the culture, while a small proportion had a morphology resembling alveolar structures (Figure 1). Histological assessment of H&E slides suggests our culture consists of alveolar type-2 cells (AT2) and bronchiolar epithelial cells with rare, flattened type-1 cells (Figure 1). IHC for TTF-1 was positive in approximately 50% of the cells in the lung organoids (Figure 2), suggestive of club cells (formerly Clara cells), respiratory epithelium, and/or AT2 cells. In bulk RNA-seq, the lung marker, NK2 homeobox 1 (*NKX2-1*) (Dost et al., 2020), was upregulated in organoids (Figure 3B), while Surfactant Protein B (*SFTPB*) and Surfactant Protein C (*SFTPC*) gene expression were detected and specific to both lung tissues and organoids (Figure 3D). Upregulated genes in organoids compared to tissues included Pyroglutamylated RFamide peptide (*QRFP*) and Peptidoglycan recognition protein 1 (*PGLYRP1*). For lung-specific genes, intra-organoid comparison revealed 736 unique genes (Figure 4A), intra-tissue comparisons revealed 1,963 unique genes (Figure 4B), and 10,597 genes were expressed in both organoids and tissues (Figure 4C). In the lung organoids, there were ∼8,389 cells, 52,423 mean reads per cell, and 4,316 median genes per cell. A total of 3 distinct cell clusters by UMAP were identified as alveolar type II cells, stressed alveolar cells, and cycling epithelial cells (Figure 6A). Alveolar type II cells had high expression of *SFTPC* and *FILIP1* (Figure 6B).

#### Urinary bladder

Urinary bladder organoids displayed a single phenotype constituting round organoids without a visible lumen or internal chamber typical of cystic organoids of other tissues (Figure 1). H&E-stained organoids had morphological features consistent with transitional epithelium, consisting of basal and umbrella cell layers (Figure 1). While IHC of UPKIII did not identify umbrella cells in the urinary bladder organoids during initial growth (Figure 2), it was expressed after thawing of the line (Supplementary Figure S4), potentially due to the additional maturation time. Desmoglein 3 (*DSG3*), and Loricrin cornified envelope precursor protein (*LORICRIN*), were upregulated in the bulk RNA-seq analysis of organoids compared to tissues (Figure 3A). Uroplakin 2 (*UPK2*) was also upregulated in organoids (Figure 3B). The bladder-specific marker *EIF4A1* was expressed in both organoids and tissues. Regarding bladder-specific genes, intra-organoid comparisons revealed 1,071 unique genes (Figure 4A), intra-tissue comparisons revealed 949 unique genes (Figure 4B), and 10,499 genes were expressed in both organoids and tissues (Figure 4C). In the urinary bladder organoids, there were ∼8,308 cells, 41,432 mean reads per cell, and 3,341 median genes per cell. A total of 3 distinct cell clusters by UMAP were identified as basal cells, umbrella cells, and cycling epithelial cells (Figure 6A). Basal cells had high expression of *LYPD3* and *RECK*, while umbrella cells had high expression of *UPK1A* and *UPK2* (Figure 6B).

#### Liver

Liver-derived organoids morphologically resembled the pancreatic-derived organoids, with one phenotype resembling a cystic morphology and another of a flowering organoid. Cellular morphology with H&E staining suggests that most of the cells were differentiated cholangiocytes (Figure 1). Hepatocyte specific antigen (HSA) stained hepatocytes were in the liver tissue while none were in the organoids (Figure 2). A single organoid exhibited a unique morphology compared to other organoids; however, organoids with this morphology were not in additional sections stained with HSA and could not be evaluated. Trefoil factor 1 (*TFF1*) and Tripartite Motif Containing 71 (*TRIM71*) were upregulated in liver-derived organoids compared to liver tissue (Figure 3A). *TRIM71* was also upregulated in organoids (Figure 3B). Liver-specific organoid markers included multiple heterogeneous nuclear ribonucleoproteins (Figure 3D). Albumin (*ALB*) was the most highly expressed liver-specific gene in tissue due to the large percentage of hepatocytes (Figure 3D). Regarding liver-specific genes, intra-organoid comparisons revealed 1,858 unique genes (Figure 4A), tissues had 1,386 unique genes (Figure 4B), and 9,984 genes were expressed in both organoids and tissues (Figure 4C).

### Insights into organ-specific genes

The usage of bulk RNA-seq allowed for identification of differentially expressed genes between tissues and organoids (Figure 3A), and scRNA-seq assisted in the determination of the major cell populations present and absent in the organoid cell lines (Figure 6). We acknowledge that the organoids are exclusively composed of epithelial cells and lack other populations present in intact tissue, such as immune cells and endothelial cells. Genes expressed were identified for each tissue type (Figure 4C) to emphasize the similarity of expression patterns of the organoid models compared with their tissue of origin. A comparison of mRNA expression across tissues and organoids can be seen in Figure 4. Between 76.4% and 80.9% (Figure 4C) of all expressed genes overlapped for each organ between tissues and organoids. This highlighted the similarity of genes present in our epithelial model, but not necessarily their expression level. Furthermore, to determine whether the genes correlate with functional outcomes, protein analysis should be completed.

Principal component analysis (PCA) (Figure 4D) was used to visualize major sources of genetic variance for the different samples, where principal components 1 and 2 (PC1, PC2) effectively separated epithelial organoids and their tissues of origin. Furthermore, PCA across all organoids and all tissues, separately, clearly demonstrated the strongest sample clustering between genetically related animals (Figure 4E). Intra-organoid and intra-tissue comparisons identified upregulated genes (Figures 3B,C) and the ten most highly expressed tissue-specific genes (Figure 3D). The expression of unique and overlapping genes was further compared for each sample type (Figures 4A,B). Overall, when comparing pathways, tissue and organoid samples were best separated by transcriptional hallmarks associated with inflammation and proliferation, respectively (Figure 5D). Stouffer integration of transcriptional pathway z-scores yielded a consensus scoring of the major global differences between paired organoids and source tissues (Figure 5B, Supplementary File S7). When comparing the scRNA-seq data of the four organoid lines to each other, the relation of cell type lineages was seen (Figure 6A). Furthermore, in all four scRNA-seq samples, clusters related to cycling cells were observed. This shared expression of certain cycling genes includes *STMN1* and *BUB1*. This data assists in determining potential applications for these novel canine organoid models (Figure 5C).

## Discussion

### Canine organoids as biomedical models

Canines can serve as a superior model to mice for translational research applications, especially due to their tendency to develop analogous chronic diseases to those of humans and their shared similarity in lifestyle (Schiffman and Breen, 2015). However, using canines for translational research presents some obstacles. Their use in research can be ethically questionable and resource-intensive. Organoids can overcome some of these challenges and could potentially represent an excellent alternative to expanding the biomedical applications of the canine model. Developing novel canine organoid models will accelerate research efforts toward advanced veterinary therapeutics as well as for preclinical drug screening in human medicine.

Many laboratories utilize tissue-specific protocols and media supplemented with various growth factors to expand their organoids which can be costly and laborious (Kaushik et al., 2018; Tortorella et al., 2022). In the current study, all described cultures were grown under the same conditions using the same organoid expansion media (*Complete media with growth factors with ROCK inhibitor and GSK3β inhibitor–CMGF+ R/G*) and differentiation media (*Complete media with growth factors–CMGF+*). The use of the same media composition lends itself to future applications of co-culture or use in assembloid models (Paşca et al., 2022), where multiple organoid lines are combined and continued growth in a shared media composition is ideal. While our media composition allows for the growth and expansion of organoids and a variety of cell types, the authors acknowledge that for differentiation of certain epithelial cell types, specific growth factors and culture conditions may need to be optimized per tissue, to support or expand cell types such as insulin-producing β cells or hepatocytes. Additionally, many protocols emphasize tissue removal to create a suspension of stem cells during organoid isolations. Here, we utilize mechanical dissociation during isolation for the inclusion of small tissue pieces during the initial growth, similar to the EnBloc method (He et al., 2020), as opposed to the single-cell digestion method. We suggest the inclusion of tissue can benefit canine organoid expansion, which seemed to assist liver cultures the most. The tissue is then dissociated enzymatically during organoid passages and hence removed from the culture prior to any analysis. This phenomenon we observed may be due to intercellular signaling from the stem cells still attached to damaged tissue, resulting in the release of damage-associated signals, increasing the initial growth of the stem cells, or potentially due to the availability of the extracellular matrix from the tissue at the beginning of the culture.

We report the establishment, characterization, and comparison of six organoid lines (endometrium, pancreas, kidney, lung, urinary bladder, and liver), one not previously reported, derived from the same animal, from two genetically related canine donors of the same litter, sex, and age. Additionally, the isolation, cultivation, and media composition were identical for the six organoid cell lines, eliminating the need for tissue-specific growth factors. To the best of the authors’ knowledge, the data in this study, therefore, constitutes the most comprehensive comparison of tissue-specific expression across canine organoids available to date. These newly available canine organoids could be applied for more rapid translational applications, such as the identification of new therapeutics, the study of genetic editing technologies, and the development of improved disease models. Due to the nature of the samples being derived from the same donors, these lines have the potential to be used in downstream experiments including organ-on-a-chip (Dongeun et al., 2010) and assembloid cultures (Birey et al., 2017). Furthermore, the similarity seen between the organoids derived from two related donors regarding morphology and transcriptomic expression supports the reproducibility of the methods. While the transcriptomic expression did not show similar top genes in the tissue and organoids, we emphasize that these types of comparisons highlight major cell populations and the complexity of tissue (including immune and stromal cells) lead to inadequate assessment of the epithelial model. Furthermore, we are planning work to utilize scRNA-seq data to identify druggable targets in our models. Future work should investigate how much variation is present across healthy organoids derived from donors of various ages, breeds, and sexes.

### Organoid characterization and biomedical applications

#### Uterus

*UPK1B* was upregulated in our canine endometrial organoids and was also found to be upregulated after local injury (endometrial biopsy) in humans where the protein was localized in glandular-epithelial cells (Kalma et al., 2009). Human endometrium organoids typically resemble a cystic-shaped organoid unlike the canine endometrial organoids, which contained large tubular structures (Boretto et al., 2017; Turco et al., 2017). One study administered hormones to the culture and noticed columnar epithelial morphology with the formation of larger vacuoles (Cindrova-Davies et al., 2021). Previously, cultures of 3D uterine glands explants and stromal cells had limited viability surviving only for 4 days with the resemblance of spheroids beginning to form (Stadler et al., 2009). *SOX17* was upregulated in our organoids, with the protein being expressed in human endometrium, specifically in the luminal and glandular epithelium (Kinnear et al., 2019). *SOx17* is important for endometrial glandular development and function in mice (Turco et al., 2017), and was also expressed in organoids derived from human menstrual flow, consistent with their function in endometrial gland development (Cindrova-Davies et al., 2021). Furthermore, 3D organotypic canine endometrium cultures have been previously described (Bartel et al., 2013), however this study simply isolated differentiated endometrial glands and stromal cells from tissue and co-cultured them for 48 h, not attempting to proliferate or expand the cells. Endometrium organoids may also be useful in the future for investigating endometrial cancers (Turco et al., 2017). Additionally, co-culturing endometrium organoids on three-dimensional scaffolds may provide insight into implantation studies (Rawlings et al., 2021; Cindrova-Davies et al., 2021).

#### Pancreas

The characterization of our canine pancreas-derived organoids suggests that they primarily contain pancreatic ductular cells. *NKX6-1* was expressed in the organoids and serves as a marker of multipotent pancreatic progenitors, indicating their ability to differentiate into ductal, acinar, and endocrine cells (Wiedenmann et al., 2021). We believe these stem cells should have the capacity to differentiate into neuroendocrine cells but optimization of growth factors in the media may be required to increase this population. The expression of *KRT7* indicates that most of the cells in the organoids are of epithelial origin and likely represent pancreatic ductal cells (Wiedenmann et al., 2021). In dogs, pancreatitis is by far the most common disease of the exocrine pancreas (Lim et al., 2014; Xenoulis, 2015). Therefore, a healthy pancreatic canine organoid model could assist in studying the pathophysiology of pancreatitis in this species. Murine *in vivo* models have long been known to have limited translatability for modeling pancreatic cancer in humans (Bailey and Carlson, 2019). Canine pancreatic ductal organoids could potentially be used for disease modeling of pancreatic ductal adenocarcinoma (PDAC), which is one of the most lethal types of cancer in humans (Holokai et al., 2020; Wiedenmann et al., 2021). Additionally, further differentiation of our canine pancreatic cultures can be attempted in the future since recently described methods successfully differentiated 2D canine pancreatic ductal cells into insulin- and glucagon-producing beta-like cells (Gao et al., 2022). Such applications could then be used for pancreatic hormone production and studying drug target screening and toxicological effects on the endocrine pancreas.

#### Kidney

Madin-Darby canine kidney (MDCK) cells have been extensively used to study permeability- and efflux-transport of drugs developed for the human market (Ye et al., 2020). The MDCK model and other immortalized cell lines fail to display functional differentiation and cannot completely recapitulate the phenotype of the primary cell (Ashammakhi et al., 2018). Due to the rapid evolution of organoid technology, genetic editing of kidney organoids for disease modeling and the use of organ-on-a-chip technology hold much promise in drug development (Ashammakhi et al., 2018). Previously, adult canine kidney epithelial stem cells were grown and adhered to the bottom of plates, but these simply gave rise to dome-forming tubular organoids when transferred on top of a Matrigel dome (Chen et al., 2019). Here we describe the derivation of the 3D canine kidney organoids, with unique morphology mimicking the tissue of origin (Figure 1), which in the future, by culturing them on transwells, could be used for drug screening purposes and eventually replace the use of the MDCK system (Nishinakamura, 2019). We hypothesize the presence of UPKIII expression only after thawing the organoids to be due to either one cell type not recovering after thawing, or during the extended growth of the culture, after thawing, one cell type out competed the other. Upon closer observation of the biopsy harvested, medulla, cortex, and renal pelvis were all collected in the same sample which complicates the predicted cell populations present. In humans, adult stem cell-derived kidney organoids represent proximal tubules, loop of Henle, distal tubules, and collecting duct cells while lacking glomerular cells (Schutgens et al., 2019). While dogs and humans differ in some kidney influx and efflux transporters, many commonalities could allow canine kidney organoids to be used for drug development and as a tool for greater understanding of kidney toxicity in veterinary and human medicine (Martinez et al., 2021).

#### Lung

Canine lung organoids have previously been described (Shiota Sato et al., 2023); however, the organoid cultures we describe here contained a variety of distinct morphologies in addition to what has previously been reported. Due to the differences in the abundance of various morphologies, it was difficult to determine the expression of the least populated phenotypes using bulk RNA-seq. Nonetheless, phenotypic and scRNA-seq characterization suggest that our culture contains alveolar type 2 (AT2) cells. AT2 cells are responsible for expression of surfactant proteins in the lungs and differentiation into AT1 cells which cover more than 95% of the alveolar surface area and are crucial for gas-exchange (Barkauskas et al., 2013; Wang Y. et al., 2018). Lung organoid models have recently helped to improve our understanding of lung repair and regeneration and aided in identification of damage-associated transient progenitors (DATPs) which represent a distinct population of AT2-lineage cells (Choi et al., 2020). Having both bronchiolar epithelial cells and AT2 cell types present in our lung organoids increases the number of future potential applications of the organoids. It has been shown that the lung arises from cells expressing the NKX2-1 transcription factor (Kaushik et al., 2018), which is upregulated in our lung organoids. For example, lung organoids have previously been used in the Transwell system for studying viral uptake into cells (Zhou et al., 2018). The use of a lung organoid model derived from human pluripotent stem cells showed that AT2-like cells are susceptible to SARS-CoV-2 infection, and infection of organoids resulted in the upregulation of chemokines similar to that reported in patients with COVID-19 (Han et al., 2021). Similarly, canine lung organoids could be used for in-depth pathophysiology studies of viruses causing canine infectious respiratory disease (CIRD) complex, including canine parainfluenza virus (CPIV), canine adenovirus (CAV) type 2, and canine herpesvirus (Reagan and Sykes, 2020).

#### Urinary bladder

Since canine bladder cancer is a well-established model for human muscle-invasive bladder cancer (Knapp et al., 2020), canine bladder cancer organoids represent a valuable model for translational preclinical research (Minkler et al., 2021). In addition, Elbadawy et al. has recently described healthy canine bladder organoids (Elbadawy et al., 2022). This report expands the knowledge and accessibility of healthy canine urinary bladder organoids, with those described here displaying a similar morphological phenotype to those previously described (Elbadawy et al., 2022). Uroplakin (UPK) proteins were expressed in our organoids and are specific to terminally differentiated urothelial cells (Lu et al., 2022). Desmoglein 3 (*DSG3*), which is a basal cell marker (Elbadawy et al., 2019), and Loricrin cornified envelope precursor protein (LORICRIN), an intermediate cell marker (Lin et al., 2013), were transcriptomically expressed in the organoid cultures. Using 3D patient-derived tumor organoids to predict the response to chemotherapeutic protocols has great potential in oncological precision medicine. Therefore, there is a need for healthy canine urinary bladder organoids to serve as controls when attempting to identify novel therapeutic strategies (Yu et al., 2021).

#### Liver

*TFF1* was upregulated, which encodes a protein critical in the regeneration of the liver after injury by promoting biliary lineage differentiation and inhibiting hepatic lineage (Hayashi et al., 2018). Single-cell RNA sequencing of the human liver described a transcriptional profile of a cell population within cholangiocytes where the DE genes included *TFF1* (MacParland et al., 2018). Additionally, Trim71 has previously been hypothesized to be involved in promoting rapid self-renewal in undifferentiated mouse embryonic stem cells (Chang et al., 2012). Our group has recently further standardized the protocol for canine hepatic organoid culture (Gabriel et al., 2022). Based on the characterization outlined in that publication, our canine liver organoid culture is mainly comprised of differentiated cholangiocytes, further aligning with the results of our lines reported here and consistent with previous descriptions in other liver-derived organoid cultures (Aktas et al., 2022; Zdyrski et al., 2024). The previous study describing canine hepatic organoids in expansion media showed that the organoids minimally expressed the mature hepatocyte (*CYP3A12*) marker while stably expressing the following markers: stem cell (*CD133* and *LGR5*), cholangiocyte (*KRT19* and *SOX9*), and early hepatocyte (*FOXA1* and *HNF4α*) (Nantasanti et al., 2015). Future efforts could involve the development of media compositions, including growth factors that will enhance the differentiation of hepatic stem cells into mature, differentiated hepatocytes rather than cholangiocytes, which were first described in murine liver organoids (Huch et al., 2013), then first attempted in dogs (Nantasanti et al., 2015), and further refined in dogs (Kruitwagen et al., 2020). Our group has recently investigated the ability of canine liver-derived organoid cultures to differentiate into mature hepatocytes by comparing six different media compositions (Gabriel et al., 2024). Optimization of such differentiation media could open avenues to explore their usefulness for hepatic toxicity assays in drug research, in addition to modeling various analogous cholangiopathies and hepatocellular diseases in canines.

We believe matching scRNA-seq from the tissues of origin for all these models is a limitation of the current study and a topic that should be explored next to assist in the development of these *in vitro* models. Furthermore, advances in organoid technology are being made in areas including personalized drug testing using patient-derived organoid cultures (Huch and Koo, 2015; Luce and Duclos-Vallee, 2025; Mullenders et al., 2019) and organoid/immune cell co-culture models (Chakrabarti et al., 2021). Typically, studies report the cultivation of one tissue-specific organoid cell line, while others combine organoid lines from multiple individuals to make conclusions. By combining unrelated donors’ information, donor-to-donor variability can be neglected, thus ignoring relevant differences in patient populations. We aimed to broaden the applications of the dog model in biomedicine while minimizing animal usage by developing these previously unreported canine organoid lines and studying gene expression profiles across different epithelial tissues.

## Conclusion

Applications of organoid technologies are rapidly expanding and now encompass protocols to develop reliable *in vitro* models of various diseases. Further differentiation or enrichment of certain cell populations within the organoids characterized here is warranted to expand the current scope of applications for canine organoids. We report the successful isolation of six canine organoid lines and identify tissue-specific genes such as uroplakins for bladder expressed in the organoid cultures. These organoid lines will potentially lower reliance on *in vivo* subjects and enhance future use of the technology in fields including drug development, clinical applications, and personalized medicine applications. Furthermore, a multi-tissue comparison of six canine organoid lines derived from two genetically related individuals allowed for direct evaluation of inter-organ and inter-individual variance in both *in vivo* and *in vitro* gene expression. Future directions, including testing of their functional recapitulation of the tissue of origin is needed to validate these as representative models and will increase the applicability of these new translational *in vitro* models.

## Materials and methods

### Tissue collection

Dogs were used under permit (ref. IACUC-18-065), and proper Institutional Animal Care and Use Committee (IACUC) protocols were followed. For this study, two 4-week-old intact female canines were euthanized via intravenous sodium pentobarbital overdose due to unrelated reasons, and tissues were quickly harvested (donor details in Supplementary File S3). Approximately 2 cm × 2 cm tissue biopsies were obtained and then rinsed three times in 10 mL of 1X Complete Chelating Solution (1X CCS, composition and further details can be found in Gabriel et al., 2022) and transferred to 6 mL of Dulbecco’s Modified Eagle Medium/Nutrient Mixture F-12 (Advanced DMEM/F12) with the addition of Pen Strep (Gabriel et al., 2022).

### Organoid isolation and cultivation

Organoid isolation and maintenance were based on a modified protocol described by Saxena et al., in 2016, which was optimized to include the standardized culture, expansion, and harvesting of canine intestinal and hepatic organoids in Gabriel et al. (2022) and Saxena et al. (2016). All tissues were isolated on the same day and processed as described below. A subset of the tissue pieces was minced with a scalpel until a consistency was achieved that would fit into a 10 mL pipette, at which point the samples underwent the typical canine organoid isolation protocol. Samples were washed with 5 mL of 1X complete chelating solution (CCS) then vortexed. After the tissue settled, the supernatant was removed down to the 5 mL mark and a total of five washes were done. During the last two washes, the supernatant was removed down to the 3 mL mark. Next, 3 mL of 1X CCS containing the tissue sample was transferred to a 6 well plate, the sample tube was rinsed with an additional 3 mL of 1X CCS and transferred to the 6 well plate. Then, 150 µL of 0.5 M EDTA (Invitrogen, ref. 15575-038) was added and the plate was incubated at 4 °C while rocking for 10 min. The sample was then transferred to a tube containing 5 mL of CCS and 2 mL of FBS and inverted. Next, the supernatant and ∼100–200 µL of tissue was transferred to an empty tube and centrifuged at 700 *g* for 5 min at 4 °C. Supernatant was discarded and the sample was rinsed with 6 mL of DMEM, before spinning and removing supernatant. Samples were then mixed in either of two Matrigel compositions Phenol red-free (Corning ref. 356231) or Phenol red (Corning ref. 356230) and plated in 30 µL drops in 24 well plates. Plates were incubated for ∼20 min at 37 °C to solidify the Matrigel. Samples were expanded in our growth media (CMGF+ R/G), which is supplemented with Y-27632 ROCK inhibitor (Biogems, ref. 1293823) and a GSK3β inhibitor, Stemolecule CHIR99021 (Stemgent, ref. 04-0004). The same media was used for all organoid lines and the media composition for the initial growth is listed in Supplementary File S2 and consists of growth factors which encourage the growth of multiple tissues. Additionally, the media composition had evolved across the 3 years and the media used for expansion after thawing for scRNA-seq is also listed in (Supplementary File S2) along with the usage of Matrigel Matrix for Organoid Culture (Corning; 356255). Total media volumes typically consisted of 500 µL on Monday and Wednesday, and 750 µL on Friday. Passaging was done as previously described, through the addition of 500 µL TrypLE Express to 500 µL of DMEM and organoids which was then incubated in a 37 °C heat bath for 10 min. Dissociation was stopped by dilution of the TrypLE Express with 6 mL of DMEM which was then centrifuged and removed. Cleaning of the organoids was used to replace Matrigel, change the density of the organoids, or help remove fibroblast-like cells from the culture. Samples were passaged between two and four times to remove excess tissue fragments before being harvested for characterization and freezing (Supplementary Figure S1). Wells with any remaining tissue were excluded from any characterization to ensure reliable results. Before harvesting for characterization, both Y-27632 ROCK inhibitor and GSK3β inhibitor were retracted (CMGF+) for 5 days to discourage the culture from stem cell expansion and allow time for potential differentiation of cell lines (see Supplementary File S2 for complete media details). Samples used for paraffin embedding were ensured to be plated in Phenol red-free Matrigel.

### Cryopreservation of organoids

Two freezing medias were used to cryopreserve organoids which consisted of (1) 50% CMGF+ R/G, 40% FBS, and 10% DMSO as well as (2) Cryostor CS10 (BioLife Solutions; 210102). Recovery after Cryostor CS10 was more reliable and thus was favored. Prior to freezing, organoids were recovered from Matrigel and resuspended in an appropriate freezing media. After being placed in a 1 mL cryovial, samples were placed in the fridge for 10 min, then moved to a −80 °C freezer overnight in a Mr. Frosty container (Nalgene; 5100-0001) filled with isopropanol and finally stored in liquid nitrogen (−196 °C) indefinitely.

### RNA extractions and bulk RNA sequencing

After isolation and expansion (between 17 and 31 days), organoids were pelleted and resuspended in 100 µL of Phosphate Buffered Saline (PBS) and transferred to a cryovial. The sample tube was flushed with 900 µL of RNAlater and subsequently added to the cryovial before being stored in liquid nitrogen (−196 °C). Tissue biopsies were directly placed into cryovials containing 1 mL of RNAlater and stored in liquid nitrogen. Upon thawing, tissue samples were quickly rinsed in PBS to remove excess salts from the RNAlater solution and were immediately transferred to 800 µL of Trizol and homogenized with a pestle. Organoid samples were thawed and transferred to a 15 mL tube with 2 mL of PBS, then centrifuged at 1,200 g at 4 °C for 5 min to pellet the organoids. RNAlater was removed, and 1 mL of Trizol was added to the organoids and homogenized via brief vortexing.

After homogenizing, samples were stored at room temperature for 5 min and then centrifuged at 12,000 g at 4 °C for 10 min to eliminate debris and polysaccharides. The supernatant was transferred to a new tube, and chloroform (0.2 mL chloroform per mL Trizol) was added. Samples were shaken vigorously for 20 s and stored at room temperature for 2–3 min before being centrifuged at 10,000 g at 4 °C for 18 min. The aqueous phase was transferred to a sterile 1.5 mL RNase-free tube. Then an equal volume of 100% RNA-free EtOH was slowly added and mixed before being transferred to a Qiagen RNeasy column (RNeasy Mini kit) seated in a collection tube which was centrifuged for 30 s at 8,000 g. Flow-through was discarded, and the Qiagen DNase treatment protocol was followed. Next, 500 µL of buffer RPE was added and centrifuged for 30 s at 8,000 g. Flow-through was again discarded, and 500 µL of buffer RPE was added and centrifuged for 2 min at 8,000 g. Flow-through was discarded, and columns were centrifuged for 1 min at 8,000 g to remove the remaining buffer. RNA was eluted in 50 µL of RNase-free water and allowed to sit for 2 min before being centrifuged for 1 min at 8,000 g. Samples were centrifuged again at 8,000 g, immediately analyzed on a Nanodrop, and frozen at −80 °C.

Prior to library preparation, RNA samples were quantified with an Agilent 2100 Bioanalyzer (Eukaryotic Total RNA Nano). Further quantification was done by GENEWIZ using a Qubit 2.0 Fluorometer (ThermoFisher Scientific) and a 4200 Tapestation (Agilent). An ERCC RNA Spike-In Mix kit (ThermoFisher Scientific cat. 4456740) was used to normalize total RNA prior to library preparation. A NEBNext Ultra II RNA Library Prep Kit for Illumina (New England Biolabs, Ipswich, MA, United States) was used for library preparation. mRNAs were initially enriched with Oligod(T) beads and then fragmented for 15 min at 94 °C. Next, first and second-strand cDNA was synthesized, end-repaired, and adenylated at 3′ends, and universal adapters were ligated to cDNA fragments. This was followed by index addition and library enrichment by PCR with limited cycles. Libraries were validated on the Agilent TapeStation (Agilent Technologies, Palo Alto, CA, United States) and quantified using a Qubit 2.0 Fluorometer (ThermoFisher Scientific, Waltham, MA, United States) as well as by quantitative PCR (KAPA Biosystems, Wilmington, MA, United States). The libraries were multiplexed and clustered onto two flowcells and were loaded onto an Illumina HiSeq 4000 instrument. The samples were sequenced using a 2 × 150 bp Paired-End (PE) configuration. The HiSeq Control Software (HCS) conducted image analysis and base calling. Raw sequence data (.bcl files) generated from Illumina HiSeq was converted into fastq files and de-multiplexed using Illumina bcl2fastq 2.20 software with one mismatch allowed for index sequence identification.

### Processing, mapping, and quantification of bulk RNA-seq libraries

The total number of reads from tissues and organoids ranged from ∼18 × 10^6^ to 27 × 10^6^ (Supplementary File S4). The average Phred quality score was 35 before quality control procedures. Comparisons were made between organoids and their native tissues, across organoids, and across tissues, for both B816 and B818 individuals.

Raw sequence files were inspected in FastQC v0.11 and MultiQC v1.7 (Kim et al., 2015; Li et al., 2009) to verify their quality. Barcodes were trimmed from reads and reads with a quality score <20 were discarded from downstream analysis cutadapt v3.5 (Martin, 2011). The data set was de-duplicated with BBDuK v38.94 (https://sourceforge.net/projects/bbmap/) with a search k-mer size of 18 bp. The resulting reads were passed to SortMeRNA v2.1 (Kopylova et al., 2012) to filter out rRNA sequences based on similarity with the SILVA v111 and Rfam v11.0 databases (Gardner et al., 2009; Quast et al., 2013). After each step, reads were inspected with FastQC and MultiQC to ensure the quality of the data. Prior to the alignment of reads to a dog genome with STAR v2.5, an index was created from ROS_Cfam_1.0 (RefSeq: GCF_014441545). In average for all samples, 90.5% of the reads mapped to unique targets within the reference genome (Supplementary File S4). Sequences from the ERCC spike-in controls were included in this index to quantify their abundances in the samples. The resulting BAM files were passed to Subread v1.6 to obtain gene-level counts via the featureCounts algorithm.

### Differential gene expression analysis

Gene counts mapped to ERCC spike-in controls by featureCounts were extracted. Then, we calculated library size scaling factors based solely on ERCC counts using edgeR v3.36 (Robinson et al., 2009) as implemented in R v4.1 (Team, 2013) and using the trimmed mean of M-values (TMM) method (Robinson and Oshlack, 2010) to normalize the ERCC counts. The scaling factors were used to normalize the gene counts and calculate log2-transformed counts per million (CPM) after adding a 0.5 as a constant to all the values.

Our goal was to detect differences in gene expression between extracted tissues and the corresponding organoids. Prior to differential gene expression analysis, visualization of transcriptional variance was explored with principal component analysis and multidimensional scaling. This unsupervised analysis revealed similarity between samples as expected by tissue-of-origin (endometrium/uterus, lung, pancreas, urinary bladder, or liver) and type (extracted or organoid). No obvious outliers were detected during this exploratory analysis. The model under testing was expr = β_1_ + β_2_ x organoid, with type indicating if the sample was an organoid or not. Gene-wise dispersions were estimated, and outlier effects were reduced with the estimateDisp function in edgeR (using the robust = T option). Negative binomial generalized linear models (GLM) were fitted for each gene, and statistical significance for the difference in mean expression was obtained by performing Bayes quasi-likelihood F-tests (glmQLFTest function in edgeR). Visualization of the results via heatmaps and Venn diagrams were generated via the ComplexHeatmap (Gu et al., 2016) and VennDiagram (Chen and Boutros, 2011) R packages. Genes unique to each organ are listed for organoids in Supplementary File S5 and for tissues in Supplementary File S6. Transcriptional Pathways for both tissues and organoids were analyzed by conducting single-sample Gene Set Enrichment Analysis (ssGSEA) using the VIPER algorithm (Alvarez et al., 2016). This analysis focused on 50 Hallmark gene sets which were obtained from the Molecular Signatures Database (MsigDB) (Liberzon et al., 2015). Scaled pathway enrichment scores were converted to z-scores and was visualized and clustered using the gplots R package. All data and analysis code has been made publicly available on a GitHub repository.

### scRNA-seq harvesting

Samples were expanded after thawing in CMGF + R/G and directly harvest as opposed to removing the R/G. Media was removed from wells and 500 µL of Cell Recovery Solution was added to each well, vigorously pipette mixed, and transferred to a 15 mL tube on ice for 20 min. The tube was then spun at 100 × g for 5 min at 4 °C and the supernatant was removed down to 500 µL. Then 1 mL of TrypLE Express was added to the sample, mixed, and the tube incubated in a 37 °C heat bath for 8 min and shaken halfway through to mix the sample. After the incubation, the sample was pipette mixed to assist in dissociation of the organoids. Next, 7 mL of cold DMEM was used to stop dissociation. The sample was then strained through a pre-wet 40 µm filter and spun again. The sample was resuspended in 1 mL of DMEM and counted on an Invitrogen Countess 3 Automated Cell Counter. The sample was again spun, supernatant was removed, the pellet was resuspended in Cryostor CS10 and placed in a cryovial. The vial was immediately placed in a Mr. Frosty, incubated in a 4 °C fridge for 10 min, and transferred to a −80 °C for at least 24 h. The samples were then either placed in liquid nitrogen for storage or shipped on dry ice.

### scRNA-seq cell counting

For single-cell samples, cryopreserved single-cell suspensions were received at Azenta, South Plainfield, NJ, United States of America in dry-ice and stored in liquid nitrogen upon receipt until further processing. Live cell count was assessed using the Nexcelom Cellaca Cell Counter, in accordance with manufacturer’s protocols. Samples with sufficient cells and viability >70% were diluted and loaded onto the Chromium Controller.

### 3′ RNA library preparation and sequencing

Single-cell RNA libraries were generated using the Chromium Single Cell 3′ kit (10X Genomics, CA, United States). Loading onto the Chromium Controller was performed to target capture of ∼6,000 GEMs per sample for downstream analysis and processed through the Chromium Controller following the standard manufacturer’s specifications. The sequencing libraries were evaluated for quality on the Agilent TapeStation (Agilent Technologies, Palo Alto, CA, United States), and quantified using Qubit 2.0 Fluorometer (Invitrogen, Carlsbad, CA). Libraries were quantified using qPCR (Applied Biosystems, Carlsbad, CA, United States) prior to loading onto an Illumina NovaSeq XPlus instrument. The samples were sequenced at a configuration compatible with the recommended guidelines as outlined by 10X Genomics. Raw sequence data (.bcl files) were converted into fastq files and de-multiplexed using the 10X Genomics’ cellranger mkfastq command.

### Analysis of single-cell RNA sequencing data and clustering

Read quality was assessed using FastQC (Andrews, 2010) and high-quality reads were mapped to the canine reference genome (ROS_Cfam_1.0, GCF_014441545.1). CellRanger was used to conduct read alignment and gene expression counts for single-cell RNA sequencing (Zheng et al., 2017). Single-cell RNA sequencing data was normalized and log transformed using R package Seurat (Butler et al., 2018; Hao et al., 2021; Hao et al., 2024; Satija et al., 2015; Stuart et al., 2019). R and corresponding R scripts were then used for data preparation procedures, analysis procedures, and visualization (R Core Team, 2021). Data was stored on GitHub, and Zenodo was used to create Digital Object Identifiers (DOIs) for publication citations.

### Paraffin embedding and immunohistochemistry

After organoids were expanded prior to freezing, they were then allowed to grow in CMGF+ for 5 days, media was removed, and 500 µL of Formalin-acetic acid-alcohol (FAA, composition in Gabriel et al., 2022) was added to each well (Gabriel et al., 2022). After 24 h, FAA was replaced with 70% ethanol and samples were paraffin-embedded and mounted on slides at the Iowa State University Histopathology laboratory. Tissues were fixed in paraformaldehyde and paraffin-embedded according to standard histology procedures. Tissues and organoids were stained with hematoxylin and eosin.

For immunohistochemistry at Iowa State University, samples were deparaffinized and rehydrated through a series of alcohol changes to deionized water. Endogenous peroxidase within the samples was then quenched using a hydrogen peroxide bath. Heat induced epitope retrieval was performed using either a tris-EDTA or citrate buffer. Immunohistochemistry (IHC) antibodies for Pan cytokeratin (Agilent, M0821), and smooth muscle actin (BioGenex, MU128-UC) were used on both tissues and organoids. An indirect method of IHC staining was then carried out using a biotinylated secondary antibody followed by a streptavidin. The samples were then incubated with NovaRED™ (Vector, SK-4800) chromogen, counterstained with hematoxylin, and dehydrated. Light microscopy images were taken on a Leica Aperio GT 450 Scanner and analyzed with ImageScope (v12.4.3.5008) or on an Olympus BX40 light microscope.

At the University of Georgia after thawing, samples were exclusively grown in CMGF+ R/G. Heat induced epitope retrieval for immunohistochemistry consisted of a pressure cooker set at 110 °C for 15 min using the following buffers: pH 9.0 buffer (HER2), pH 6.0 citrate buffer (TTF1), or Reveal (RV1000M, Biocare Medical) decloaker (HAS). Proteinase K antigen retrieval was used for insulin. Then the following antibodies were incubated for 1 h at room temp (except insulin which was 30 min): HER2 (1:100, AB214275, Abcam), TTF-1 (1:500, 343M-96, Cell Marque), Insulin (1:2000, I8510, Sigma), and HSA (1:1000, CM166A, Biocare Medical). At Cornell University, UPKIII (1:20, 10R-U103AX, Fitzgerald) was run through their standard protocol. DAB chromogen with hematoxylin counterstain was used for all antibodies. Images were taken with an Olympus BX41 microscope and BioVID 4k camera using ToupView software (LW Scientific, version x64 4.11.23945.20231121). Adobe Photoshop 2025 (release 26.10.0) was used to apply white balance, contrast, and saturation adjustments to whole images.

## Data Availability

The bulk RNA-seq raw reads generated in this study are available in the Sequence Read Archive (NCBI-SRA BioProject PRJNA847879) as well as the aligned files being available on the NIH ICDC (https://caninecommons.cancer.gov/#/study/ORGANOIDS01). The scRNA-seq raw reads are available on the GEO (GSE293701). The bioinformatic scripts are available on Github (https://github.com/chris-zdyrski/Novel_Canine_Organoids) and Zenodo (DOI: 10.5281/zenodo.15131584).
