# Supplementary figures and images for: Establishment and transcriptomic characterization of canine organoids from multiple tissues
Source: Front Cell Dev Biol. 2025 Nov 24;13:1680376. doi: 10.3389/fcell.2025.1680376 (PMC12686805; doi:10.3389/fcell.2025.1680376)

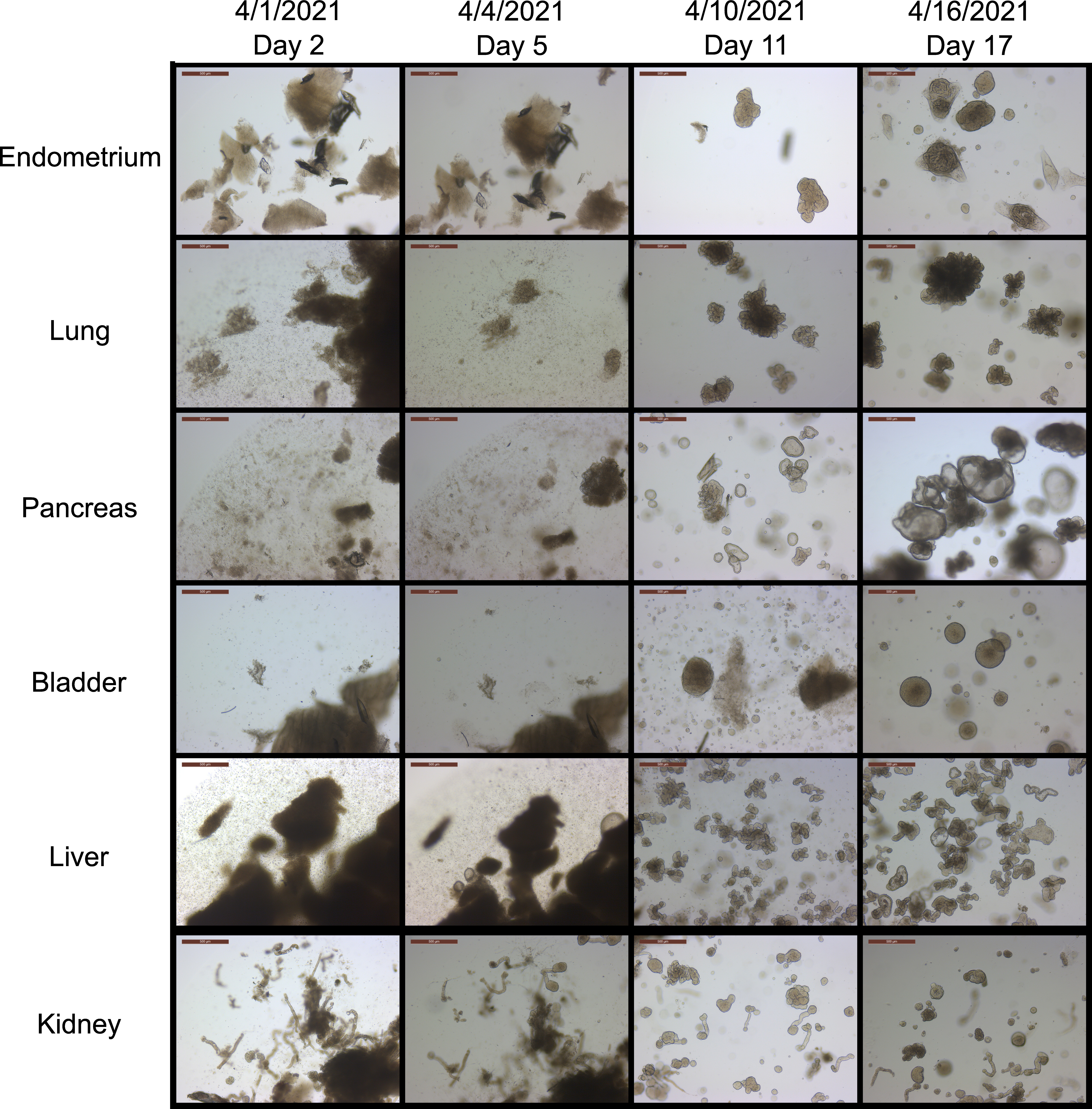

Supplement: Supplementary file 5 [file Image1.jpeg]

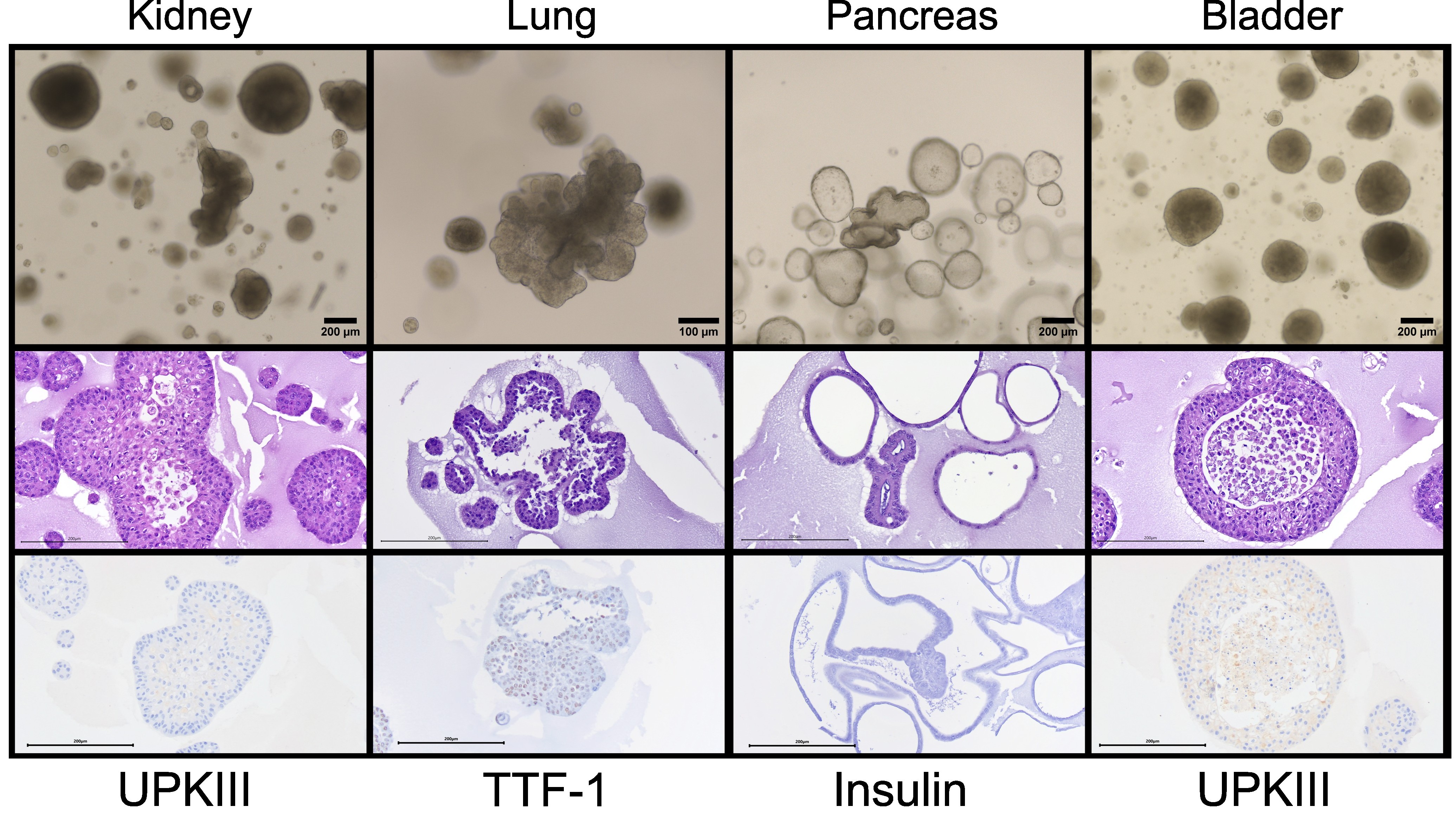

Supplement: Supplementary file 6 [file Image4.jpeg]
